# *Mycobacterium tuberculosis*

**DOI:** 10.1155/2015/857598

**Published:** 2015-08-04

**Authors:** Vishwanath Venketaraman, Deepak Kaushal, Beatrice Saviola

**Affiliations:** ^1^Department of Basic Medical Sciences, College of Osteopathic Medicine of the Pacific, Western University of Health Sciences, Pomona, CA 91766-1854, USA; ^2^Tulane University School of Medicine, Covington, LA 70433, USA

*Mycobacterium tuberculosis* (*M. tuberculosis)*, the causative agent for tuberculosis (TB), is responsible for 1.5 million deaths and 9 million new cases of TB in 2013 [1]. Lack of highly specific and sensitive diagnostic tests, restricted vaccine efficacy, emergence of multidrug and extensively drug-resistant strains of* M. tuberculosis*, and HIV coinfection are the most important factors that result in poor global TB control [1]. It is estimated that, among individuals who are infected with* M. tuberculosis*, around 85–90% are able to control the infection but are unable to completely eradicate the bacillus from their bodies resulting in a latent tuberculosis infection (LTBI) [2]. LTBI is defined by the presence of* M. tuberculosis*-specific immune response with the absence of radiological evidence of clinical disease and clinical signs or symptoms. Approximately one-third of the world's population has LTBI and, among the latently infected population, 5–10% will develop active TB due to reactivation and resuscitation of dormant bacilli [1]. This risk is further increased in immune-compromised individuals and people at the extremes of age. Latently infected individuals are the largest reservoir of potential future source of active TB [2]. In order to reduce the global incidence of TB cases, we urgently need an improved vaccine against* M. tuberculosis* infection.* Mycobacterium bovis* Bacillus Calmette-Guérin (*M. bovis* BCG), currently the only available vaccine against TB, induces variable protection in adults [3]. Immune correlates of protection are lacking, and analyses on cytokine-producing T-cell subsets in protected versus nonprotected cohorts have yielded inconsistent results [3]. This special issue encompasses findings from clinical and translational studies that will further enhance our understanding of the virulence mechanisms of* M. tuberculosis*, impaired host immune responses in individuals with active TB, pathogenesis of the disease, and strategies to enhance the innate and adaptive immune responses against* M. tuberculosis* infection.

Within this special issue are studies that explore the use of BCG vaccines that have been modified in various ways to improve their efficacy. One study uses BCG-Pasteur and BCG-China with DNA vaccine priming and boosting. The vaccine contains coding regions for antigens present within the RD-14 region absent in BCG-Pasteur. In a separate study, BCG was made as a recombinant strain with overexpression of a chimeric protein based on previously studied immunizing antigens. This chimeric antigen is controlled by the iron regulator FurA which regulates gene activity during* in vivo* growth. In another investigation BCG was constructed which overexpresses the cytokine IL-18. When dendritic cells were infected with this recombinant strain, these cells had altered responses and some immune functions were increased, such as improved cytokine production. An alternative approach to vaccination against TB infection is to create mutant* M. tuberculosis* strains that are attenuated but can still stimulate immunity. In this issue a study shows* M. tuberculosis* strain that is mutated in ferritin and is used as a vaccine in a mouse model. While this strategy was comparable to BCG with respect to bacterial burden, pathology of infection seemed to be improved.

Within this issue as well are various studies of immunological tests that can better distinguish between active TB and LTBI. One study shows that LTBI results in a distinctive CD4+ T-cell cytokine profile. Another study using analysis of antibodies against TB glycolipids was also investigated and compared with those having pulmonary TB, cavitation, and extrapulmonary disease. Also in another study comparing TB infected individuals and healthy subjects, antigen presenting cells seemed to be impaired in TB patients and more severely impaired in PPD-anergic patients.

*Vishwanath Venketaraman* *Vishwanath Venketaraman* *Deepak Kaushal* *Deepak Kaushal* *Beatrice Saviola* *Beatrice Saviola*
